# Global health electives in the COVID-19 era: resuming travel and strengthening global health academic partnerships

**DOI:** 10.5116/ijme.6272.630a

**Published:** 2022-05-31

**Authors:** Heather Haq, Amos Msekandiana, Mogomotsi Matshaba, Linoeo Thahane, Jennifer Watts, Reena Tam, Nicole St Clair, Charles Schubert, Amy Rule, Pia Pannaraj, Michael Pitt, David Oleson, Brittany Murray, Lee Morris, Joanne Mendoza, Megan McHenry, Elizabeth Keating, Kathy Ferrer, Heather Crouse, Tania Condurache, Maneesh Batra, Adelaide Barnes, James Conway

**Affiliations:** 1 Baylor College of Medicine, Baylor College of Medicine International Pediatric AIDS Initiative (BIPAI) at Texas Children's Hospital, Houston, Texas, USA; 2 Baylor College of Medicine Children's Foundation-Malawi, Kamuzu Central Hospital, Lilongwe, Malawi; 3 Botswana-Baylor Children's Clinical Center of Excellence, Baylor College of Medicine, Gaborone, Botswana; 4 Baylor College of Medicine Children's Foundation-Lesotho, Baylor College of Medicine, Maseru, Lesotho; 5 Children's Mercy Hospital, Kansas City, Missouri, USA; 6 University of Utah, Salt Lake City, Utah, USA; 7 University of Wisconsin School of Medicine and Public Health, Madison, Wisconsin, USA; 8 Cincinnati Childrens Hospital Medical Center, University of Cincinnati College of Medicine, Cincinnati, Ohio, USA; 9 Keck School of Medicine, University of Southern California, Children's Hospital Los Angeles, Los Angeles, California, USA; 10 University of Minnesota Masonic Children's Hospital, Minneapolis, Minnesota, USA; 11 University of Texas-Southwestern Medical Center, Dallas, Texas, USA; 12 Emory University School of Medicine, Atlanta, Georgia, USA; 13 Levine Children's Hospital Atrium Health, Charlotte, North Carolina, USA; 14 University of Virginia School of Medicine, Department of Pediatrics, University of Virginia Children's Hospital, Charlottesville, Virginia, USA; 15 Indiana University, School of Medicine, Indianapolis, Indiana, USA; 16 George Washington University, School of Medicine and Health Sciences, Washington, DC, USA; 17 Baylor College of Medicine, Houston, Texas, USA; 18 University of Louisville School of Medicine, Louisville, Kentucky, USA; 19 University of Washington School of Medicine, Seattle, Washington, USA; 20 The Children's Hospital of Philadelphia, Philadelphia, Pennsylvania, USA

**Keywords:** Medical education, global health, international travel, COVID-19, ethics

## Introduction

The COVID-19 pandemic has dramatically shifted medical trainees' perspectives on the importance of global public health, yet travel restrictions and safety concerns have significantly altered their opportunities to participate in site-based, in-person global health (GH) electives. GH partnerships and educational activities have, out of necessity, pivoted to the virtual space.^1^^-^^3^ The emergence of new variants continues to add complexity to decision-making and planning for international travel. As our medical education communities ponder strategies and timelines to resume international travel, we must consider the safety and ethics of resuming personnel exchanges between countries for the purposes of GH partnerships and education. This pandemic-necessitated shift in partnership practices and the pause on medical trainee travel to international elective sites offers a unique window of opportunity to reimagine GH partnerships and education in a post-pandemic world. The pandemic's magnification of health inequities, amidst broader calls for racial justice and decolonization of the field of GH,^4^^-^^8^ offers us an opportunity to reevaluate GH partnerships by centering equity and disrupting power imbalances between high-income country (HIC)-based academic institutions and partner institutions in low- and middle-income countries (LMICs).

The purpose of this perspective piece is to reflect on key considerations for HIC-based GH education programs for resuming GH electives in a safe and ethical manner while simultaneously reflecting on opportunities to strengthen GH academic partnerships. As GH educators in undergraduate and graduate medical education across the world, we do not seek to forecast a date when travel and participation in GH electives will be safe but rather suggest considerations for programs to evaluate the feasibility, safety, ethics, and timing for physical travel. We propose that there is a continued – if not greater – need for GH education in a pandemic/post-pandemic world; how GH education can be continued and reimagined in this context; and considerations for resumption of site-based, in-person GH education electives. These guidelines are likely to maintain relevancy for GH educators beyond this pandemic, as future infectious disease outbreaks are inevitable, as are other forces such as geopolitical instability, continuous human migration, climate change and weather disasters, increased air travel, and frenetic urbanization. This perspective is relevant to travelers from HIC settings such as the US, Canada, the European Union, the UK, and Australia.

## The Immediate Impact of the COVID-19 Pandemic on GH Electives

Prior to the onset of the pandemic, undergraduate and graduate medical education trainees in HICs had high levels of participation in a range of GH elective experiences across the globe. For example, recent surveys demonstrated 24.2% of US medical students participated in GH electives during medical school;^9^ 34% of German medical schools offered GH electives;^10^ up to 36% of UK medical students completed GH electives abroad;^11^ 7.3% of US pediatric residents participated in a GH elective during a given academic year, 55.5% of US pediatric residency programs offered GH electives abroad;^12^ and 47.4% of US pediatric fellowship programs offered GH electives abroad.^13^ Depending on the level of training and nature of the elective, HIC-based trainees participated in various activities during GH electives, including observation, teaching, research, and direct clinical care. For example, visiting students might observe health systems and clinical care, while visiting faculty and advanced trainees, such as fellows, may be arranged to provide critical patient care staffing at partner sites.^14^

In early 2020, HIC-based GH education programs rapidly adapted to the dynamic and unfolding COVID-19 pandemic. Policies, travel restrictions, and flight availability changed by the hour, which not only affected the planning of forthcoming electives but also how to manage trainees located at international sites. Organizations such as the Peace Corps repatriated all of their >3,000 volunteers across the globe for an unprecedented temporary suspension of activities.^15^ The heightened global travel restrictions required quick and decisive action to bring trainees back to their home countries, in some cases revealing vulnerabilities in emergency planning for GH electives. These expeditious decisions to return HIC-based trainees and medical staff home, whether voluntary or mandated by home institutions, were often unilateral and did not consider the impact on partner institutions. Many LMIC partner sites experienced sudden blows to their workforce with the rapid exodus of HIC partners. Moreover, LMIC partners were sometimes burdened with the task of supporting HIC trainee repatriation logistics at a time when their own institutions were stressed with emergency preparedness.

To our knowledge, the vast majority of HIC-based health professional trainees were relocated from GH elective sites back to their home institutions early in the pandemic and future away electives were paused, based on individual institutions' risk assessments and recommendations from professional organizations.^16^ Travel was impacted bidirectionally, and international travel of LMIC trainees and faculty was also halted during the pandemic. To adapt GH education in the absence of travel, many HIC-based programs offered trainees local GH electives addressing health inequities and initiated a variety of online GH experiences.^3^^,^^7^^,^^17^ However, the value of these online activities to both trainees and partners has yet to be fully assessed.

The rapid exodus from LMIC partner sites highlights the inherent privilege of HIC-based trainees and academic partners and exposed the uncomfortable reality that partnership is conditional. One study in Malawi demonstrated a 66% decrease in the clinical staff workforce in a large pediatric inpatient unit as US-based GH academic partners failed to meet their human resource commitments in the context of the pandemic.^18^

## Why: The Case for Global Health Education During and Beyond the Pandemic

The pandemic exposed our global interconnectedness and demonstrated that geographical borders do not contain the spread of viruses, viral variants, or misinformation. The pandemic magnified and compounded existing health inequities globally. While the final toll of the pandemic has yet to be determined, the indirect effects on preventable diseases, malnutrition, chronic care access, mental health, and adverse economic impacts will have reverberating and longstanding impacts on GH.^19^

There are many compelling reasons for HIC-based academic programs to eventually resume site-based, in-person GH electives for trainees: (1) there is a high and sustained trainee demand for GH training;^9^^,^^12^^,^^13^ (2) GH electives offer transformative experiences that encourage and empower trainees to address health inequities and disparities in their careers;^20^^-^^23^ (3) the existence of institutional GH partnerships improves both trainee and faculty recruitment;^24^ and (4) for institutions that value equity, global engagement is often a priority. Moreover, health professionals with training in GH may leverage their experiences to prepare for and respond to future pandemics and emerging diseases; to recognize and attend to inequities; and to safeguard and advocate for disadvantaged populations.

The pause in travel also offers programs an opportunity to reevaluate and recalibrate GH partnerships to address communication, power dynamics, and partnership priorities that more equitably center the needs and priorities of LMIC partners.^25^ There are opportunities to enhance shared leadership and goal-setting,^26^ promote bidirectional exchange of trainees and medical staff,^27^ and incorporate decolonization efforts.^8^

## Sustaining GH Partnerships and Education during the Pandemic and Beyond

### Continued Partnership and Communication

While some HIC-based academic institutions did maintain the physical presence of staff and faculty at LMIC partner sites throughout the pandemic, most shifted to virtual activities and support. Strengthened internet services in many LMIC locations paved the way for increased use of video conferencing platforms. GH partners continued working remotely to collaborate on grant applications, data analysis, manuscripts, and other programmatic areas. Virtual technologies were also leveraged for online office hours, roundtable discussions and panels, telehealth support of rounds, patient consultations, and other means of virtual technical assistance.^28^^-^^30^ The proliferation of webinars, often free of charge, has also allowed multilateral participation.; however, accessibility is not uniform across LMIC institutions, and many without necessary bandwidth, devices, or data plans were unable to participate equitably. While the uptake of virtual technologies represents a welcome paradigm shift in GH engagement and may likely continue post-pandemic, the shift away from in-person engagement resulted in lost opportunities important to GH partnerships: shoulder-to-shoulder mentorship, direct participation in clinical care, research, and education, face-to-face relationship-building and bidirectional programming.

### Shared Decision-Making on Timing of International Travel

As HIC-based academic institutions continue to maintain their GH partnerships, opportunities to discuss readiness for resuming in-person engagements are critical. We encourage leaders to lean into these conversations and listen first. How was the withdrawal of trainees, staff, and faculty perceived locally? What impact did these departures have on trust or feelings of betrayal within partner institutions, and is there a need to re-establish trust? Has the global partnership been maintained? Do all parties continue to see the benefit in resuming the exchange of trainees? Are alternative models of GH capacity-building more desirable to LMIC partners, for example, shifting focus away from human resource commitments from visiting trainees or medical staff and towards capacity-building of local staff? By engaging and listening, program leaders and partners will receive feedback on safe, equitable, and ethical ways to prepare for a return to international travel. As these conversations continue and at the invitation of LMIC partners, it is important that HIC-based trainees or faculty do not place unnecessary burdens on partner sites, especially where healthcare infrastructure may have been negatively impacted by the pandemic. LMIC partners' insights and perspectives on readiness to receive international trainees is critical.^1^

### Considerations for Resuming Site-Based, In-Person Global Health Electives

While it is clear that the pandemic will inform changes to what GH electives can and should be, the question of when it will be safe and ethical for trainees to re-engage with site-based, in-person GH electives remains unanswered. Programs may wish to consider a phased approach, where travel is prioritized for those with higher levels of training, longer-term experience, and stronger relationships with partners. And over time, as LMIC partners indicate increasing readiness, trainees, including fellows, residents, and health professional students, can then resume travel. Concomitant efforts should be made to resume or encourage bidirectional travel for LMIC faculty and trainees.^27^ Important ethical considerations around international travel include over-burdening GH partner sites, especially during times of health system strain in the setting of COVID-19 surges, as well as visiting trainee use of limited supplies of personal protective equipment.

Commitment to emergency and contingency planning for site-based, in-person GH electives will be critical prior to resumption of travel.^31^ Clear articulation of responsibilities for all partner organizations can prevent misunderstandings or confusion – whether related to medical care or travel for groups impacted by disruptions - in order to minimize burdens placed on LMIC partners and ensure the safety of trainees. Conversations and agreements with legal services and risk management personnel, and clarity in contractual agreements, as well as comprehensive insurance coverage have all been highlighted during this pandemic as key factors to ensure health and safety.

As HIC-based programs evaluate various challenges to international travel of trainees during and after the COVID-19 pandemic, there are numerous complex, shifting areas to consider at the GH partnership level, from the HIC program's perspective, from the GH partner elective site perspective, international travel logistics, individual traveler risks, and country-specific risks. Programs should reevaluate GH partnership dynamics in the context of the pandemic and LMIC partners' bandwidth to provide clinical services and logistical support to visiting trainees. The HIC program should evaluate institutional policies, medical evacuation, staffing (including coverage if a trainee's return is delayed due to COVID-19), and budgets to support travel-related expenses, and update pre-departure training to cover COVID-19-related topics. Programs should also evaluate GH partner site factors, including availability of personal protective equipment and infection control policies. As international travel continues to be more complicated, real-time evaluation of travel logistics should include requirements for testing, proof of vaccination status, and quarantine requirements from the departing, transit, and arrival countries as well as air carriers. Travelers should evaluate their individual risk thresholds, factoring in personal and family members' health history and vaccination status. Finally, programs and travelers should consider country-specific factors should be considered including local travel restrictions, health system strain, local epidemiology and predominant variants, testing capacity, population vaccination coverage, and ability to travel between and within countries safely. As we have seen throughout the pandemic, rules, regulations, and policies shift rapidly; therefore, programs will need to revisit these considerations frequently in resuming travel.

## Conclusions

In today's interconnected world, the COVID-19 pandemic has exposed and magnified numerous health-related inequities, highlighting the need for continued and improved GH education and academic partnerships. The pandemic has simultaneously forced short-term changes in the way HIC-based GH education is provided but also catalyzed programmatic innovations. Such challenging and historical events offer unique opportunities to reimagine GH education, enhance collaboration, and achieve equitable GH partnerships. How we respond this time can be transformative for the future of global collaborations and planetary wellness.

### Acknowledgments

The authors wish to thank the individuals who work at partnership institutions in low- and middle-income countries who make global health electives possible.

### Conflict of Interest

James H. Conway is an Investigator/Co-investigator for Sanofi-Pasteur, AstraZeneca, and Centers for Disease Control and a consultant for Moderna, Pfizer, Merck, and GSK vaccines. The other authors have no conflicts of interest to disclose.
